# The discovery of angiogenic growth factors: the contribution of Italian scientists

**DOI:** 10.1186/2045-824X-6-8

**Published:** 2014-04-01

**Authors:** Domenico Ribatti

**Affiliations:** 1 Department of Basic Medical Sciences, Neurosciences and Sensory Organs, University of Bari Medical School, Policlinico - Piazza G. Cesare, 11, 70124 Bari, Italy; 2 National Cancer Institute “Giovanni Paolo II”, Bari, Italy

**Keywords:** Angiogenesis, G-CSF, GM-CSF, EPO, FGF-2, PlGF, VEGF

## Abstract

Angiogenesis is regulated, under both physiological and pathological conditions, by numerous “non-classic” pro-angiogenic factors, including fibroblast growth factor-2 (FGF-2), vascular endothelial growth factor (VEGF), and placental growth factor (PlGF), and “non-classic” pro-angiogenic factors, including granulocyte colony stimulating factor (G-CSF), granulocyte macrophage colony stimulating factor (GM-CSF), and erythropoietin (EPO). In the context of the most important discoveries in this field, this review article summarizes the important role played by the Italian scientists in the course of the last twenty years.

## Introduction

In 1945, Algire and Chalkley were the first to appreciate that growing malignant tumors could continuously elict new capillary growth from the host [1]. In 1970s, it was widely accepted that tumors did not produce specific angiogenic proteins. In 1971, Judah Folkman isolated the first angiogenic factor, and called it “Tumor Angiogenesis Factor” (TAF). He fractioned by gel-filtration on Sephadex G100 the homogenate of a Walker 256 carcinoma and obtained a fraction with a strong angiogenic activity with a molecular weight of about 10,000 Dalton, consisting of 25% RNA, 10% proteins, 58% carbohydrates, and a lipid residue. Several other low molecular weight angiogenic factors were isolated from the Walker 256 carcinoma, capable to induce an angiogenic response in vivo when tested on rabbit cornea or chick embryo chorioallantoic membrane (CAM), and in vitro on cultured endothelial cells [2]. Subsequently, TAF was extracted from several tumor cell lines.

Starting from the discovery of TAF, other pro-angiogenic molecules have been isolated, namely basic fibroblast growth factor (bFGF)/fibroblast growth factor-2 (FGF-2), vascular endothelial growth factor (VEGF)/vascular permeability factor (VPF), and placental growth factor (PlGF). In the meantime, it has been demonstrated the angiogenic activity of non-classic angiogenic molecules, including hematopoietic cytokines, namely granulocyte colony stimulating factor (G-CSF), granulocyte macrophage colony stimulating factor (GM-CSF), and erythropoietin (EPO).

In this context, Folkman hypothesized that tumor growth is angiogenic-dependent and that inhibition of angiogenesis could be therapeutic, introducing the term anti-angiogenesis. Investigations on neoplastic transformation have focused on transformed cells and in the meantime have addressed the tumor microenvironment and documented its importance in tumor progression. The pathogenesis of most cancers, in fact, includes complex and mutual interactions affecting tumor cells, inflammatory cells and various components of the extracellular matrix. These concepts are now widely accepted and supported by experimental and clinical studies.

Anti-angiogenic agents may be divided in two major groups: indirect agents that block the expression or the activity of angiogenic molecules, or the expression of their receptors on endothelial cells, and agents able to directly affect endothelial cell function or survival.

Beginning in the 1980s, the industry began exploiting the field of anti-angiogenesis for creating new therapeutic molecules in angiogenesis-dependent diseases. Bevacizumab (Avastin) was the first angiogenesis inhibitor approved by the Food and Drug Adminstration for the treatment of colorectal cancer in February 2004, administered in combination with irinotecan, 5-fluorouracil and leucovirin; it was subsequently approved for use, in combination with cytotoxic chemotherapy, in other cancers, demonstrating an improvement in overall survival or delayed tumor progression compared to chemotherapy alone.

Here, I have summarized the fundamental contribution of Italian scientists to the discovery of the most important angiogenic factors.

### The contribution of Marco Presta to isolation of bFGF/FGF-2

In 1970s, Armelin and Gospodarowicz demonstrated that the bovine pituitary contains a potent mitogen for fibroblasts, endothelial cells and chondrocytes, with a molecular weight of 14,000-16,000 Daltons and a basic isoelectric point. This factor was named fibroblast growth factor [3-6].

In 1980s, Shing at the Children’s Hospital in Boston discovered a tumor-derived factor very similar to the agent discovered by Gospodarowicz, able to bound with such a high affinity to heparin, with a molecular weight of 14,800, which stimulated the proliferation of capillary endothelial cells in vitro, and angiogenesis in vivo in the chick CAM assay [7,8]. Amino acid sequence was determined by Esch et al. [9] and it was purified from bovine pituitary and brain [10].

**Figure 1:**
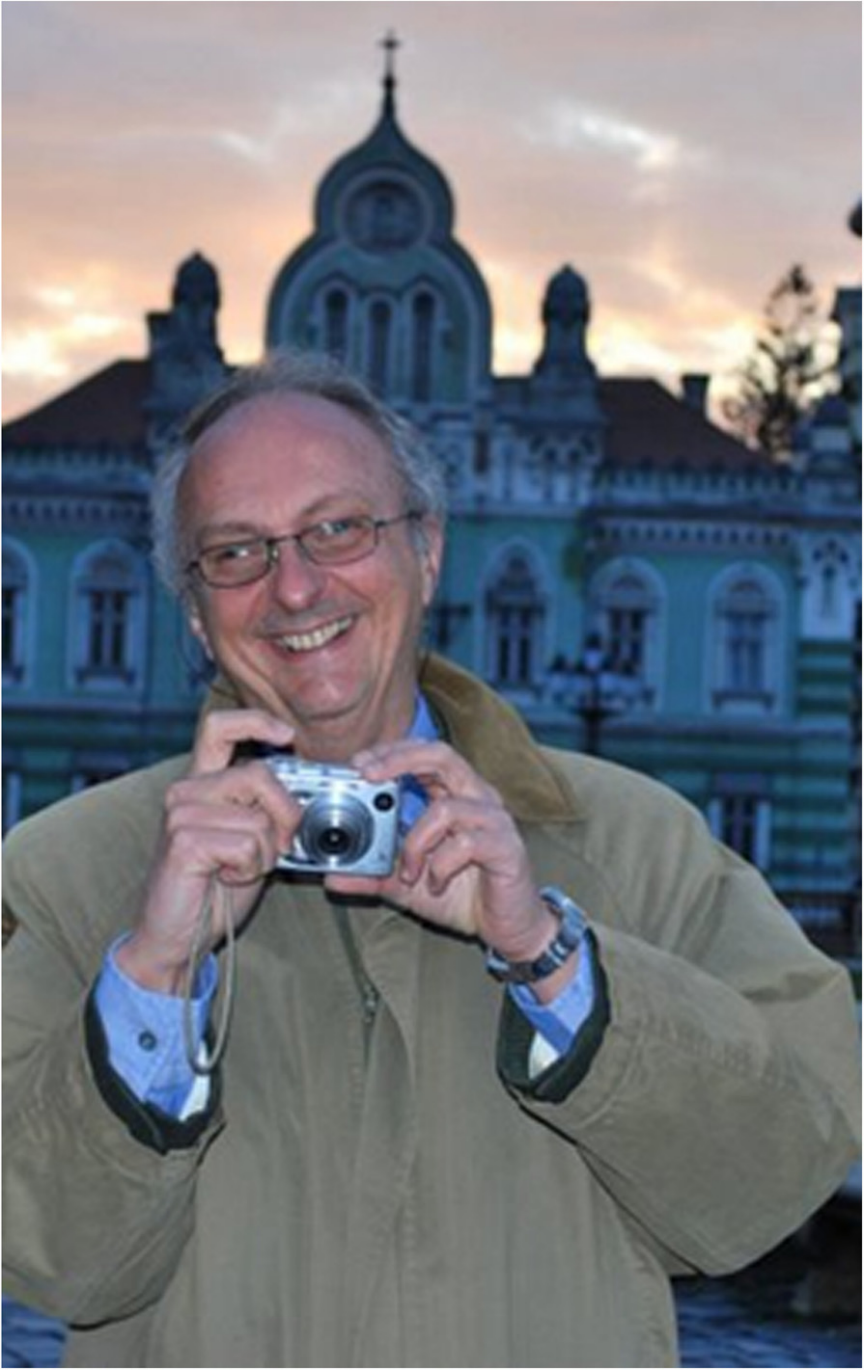
A port trait of Marco Presta.

In 1986, Marco Presta (Figure 1), Moscatelli, and Rifkin, working at the New York University, isolated an angiogenic factor from human placenta and human hepatoma cells, able to stimulate DNA synthesis, motility, and protease production in capillary endothelial cells and induced angiogenesis *in vivo*[11,12]. Amino acid sequence demonstrated that this factor was human bFGF.

### The contribution of Napoleone Ferrara to isolation of VEGF/VPF

**Figure 2:**
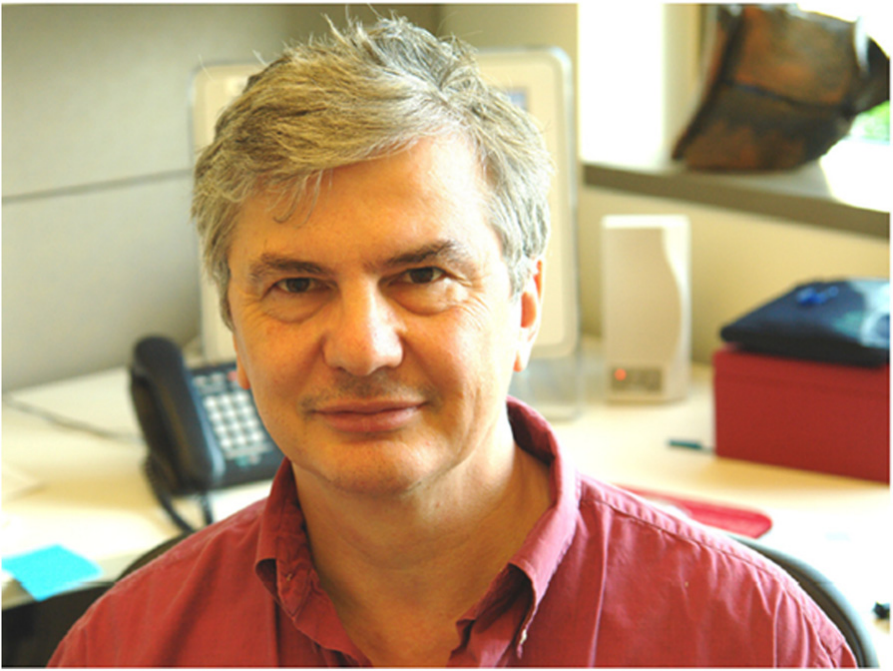
A port trait of Napoleone Ferrara.

Napoleone Ferrara (Figure 2) joined Genentech in 1988 after postdoctoral training at the University of California at San Francisco, in the Department of Obstetrics, Gynecology, and Reproductive Sciences in Richard Wiener’s laboratory, where he isolated and cultured follicular cells, a population of non–hormone-secreting cells from the anterior pituitary of cows [13]. Ferrara demonstrated that supernatants from cultures of follicular cells stimulated endothelial cells proliferation, and supposed that they secreted an angiogenic protein.

In 1989, Ferrara and Henzel isolated a diffusible endothelial cell-specific mitogen from medium conditioned by bovine pituitary follicular cells, which they named VEGF, distinct from bFGF and indeed did not match any known protein in available databases [14].

In 1979, Harold D. Dvorak, working at the Harvard Medical School in Boston, tested cell-free supernatants from several human and animal tumor cell lines in the permeability Miles assay [15], and found that they generated an intense blue spot due to extravasated Evans blue, and called this activity VPF [16]. VPF showed a potency some 50,000 times that of histamine [17,18]. The molecular cloning of VEGF and VPF revealed that both activities are embodied in the same molecule.

In 1992, in a collaborative study between Ferrara and Lewis Williams at the University of California at San Francisco, VEGFR-1 was shown to be an high-affinity VEGF receptor [19]. Moreover, Ferrara demonstrated that VEGFR-1 expression was up-regulated by hypoxia via a hypoxia-inducible factor (HIF)-1-dependent mechanism [20] and that VEGFR-1 binds not only VEGF-A, but also PlGF [21]. In 1996, Ferrara [22] and Carmeliet et al. [23] demonstrated an important role of VEGF in embryonic vasculogenesis and angiogenesis.

In 1993, Ferrara demonstrated that anti-VEGF monoclonal antibodies exerted an inhibitory effect, ranging between 70% and more than 95%, on the growth of tumor cell lines injected subcutaneously in nude mice [24]. These findings provided the first direct demonstration that inhibition of the action of an endogenous angiogenic factor may result in suppression of tumor growth in vivo. The first anti-angiogenic agent approved by the Food and Drug Administration was bevacizumab (Avastin; Genentech), a humanized version [25] of an anti-VEGF monoclonal antibody discovered by Ferrara [24]. Bevacizumab bound and neutralized all human VEGF-A isoforms and bioactive proteolytic fragments, inhibited the growth of human tumor cell lines in nude mice [25].

More recently, Ferrara has demonstrated that related endocrine gland-VEGF (EG-VEGF) and Bv8 proteins, also known as prokineticin 1 (Prok1) and prokineticin 2 (Prok2), induce both angiogenesis and haematopoietic cell mobilization [26].

### The contribution of Graziella Persico to isolation of PlGF

**Figure 3:**
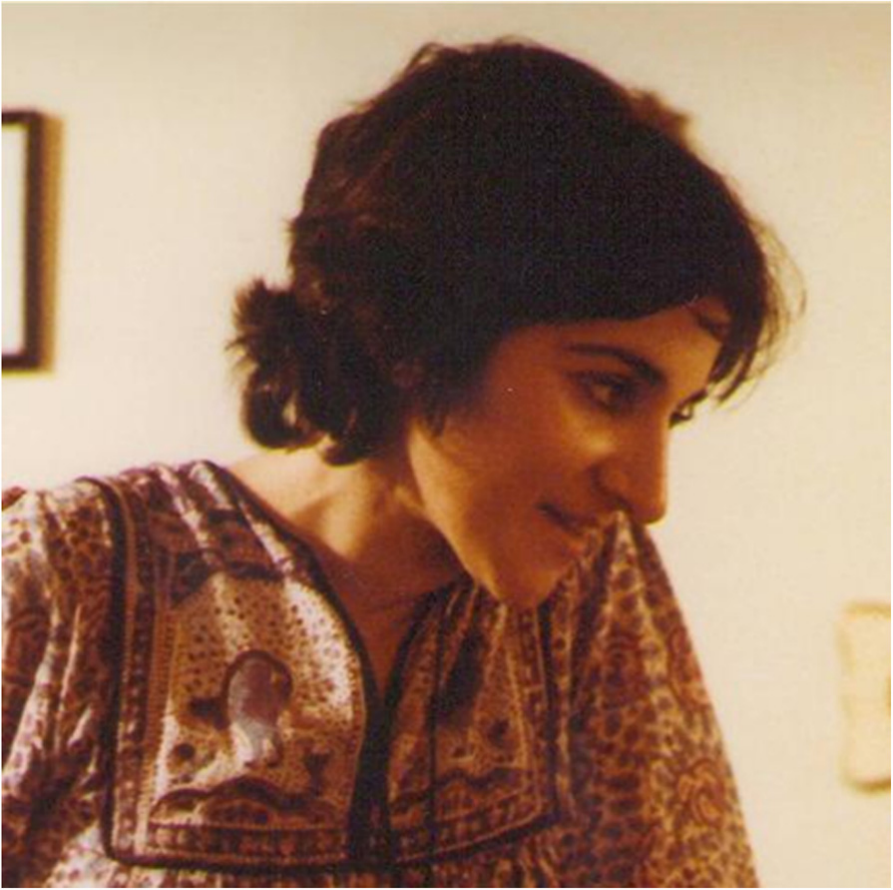
A port trait of Maria Graziella Persico.

Maria Graziella Persico (Figure 3), working at the Institute of Genetics and Biophysics in Naples, cloned and purified PlGF and determined its structure [27,28]. PlGF exhibited remarkable structural similarities to VEGF-A, although PlGF and VEGF-A showed only a 42% amino acid sequence identity, as well as significant functional differences. Persico demonstrated also that the human PlGF gene is located on chromosome 14 and consists of seven exons [29].

PlGF was originally identified in the placenta, where it has been proposed to control trophoblast growth and differentiation [29,30], playing a role during invasion of the trophoblast into the maternal decidua [31]. Ziche et al. [32] demonstrated that PlGF-1 was angiogenic in vivo in the rabbit cornea and in the chick CAM. PlGF and VEGF were able to induce the formation of new capillaries approximately 36 h earlier than FGF-2, suggesting a faster recruitment of endothelial cells.

In 2001, Persico in collaboration with Carmeliet, showed that loss of PlGF does not affect development, reproduction or postnatal life [33]. However, in ischemic conditions such as myocardial infarction or following ligation of the hind limb artery, PlGF−/− mice showed reduced angiogenesis and arteriogenesis [33]. Administration of recombinant human PlGF was able to reverse the revascularization defect within the infarcted area. VEGF-A and VEGFR-2 expression remains at comparable levels in PlGF−/− mice, in which the loss of PlGF blocks the recruitment of macrophages, suggesting that PlGF has a specific role in macrophage mobilization [33].

As concerns the effect of an anti-PlGF antibody, even if evidence has shown that it exerts an anti-angiogenic action, Bais et al. [34] demonstrated that although anti-PlGF treatment inhibited wound healing, extravasation of tumor cells cells, and growth of a tumor overexpressing the PlGF receptor (VEGFR-1), neutralization of PlGF using blocking antibodies had no significant effect on tumor angiogenesis in different animal models.

### Angiogenic activity of classical hematopoietic cytokines

G-CSF and GM-CSF can promote proliferation and differentiation of myeloid-committed progenitors, including eosinophils, basophils, megakaryocytes, and erythroid and dendritic cells in synergy with other factors.

**Figure 4:**
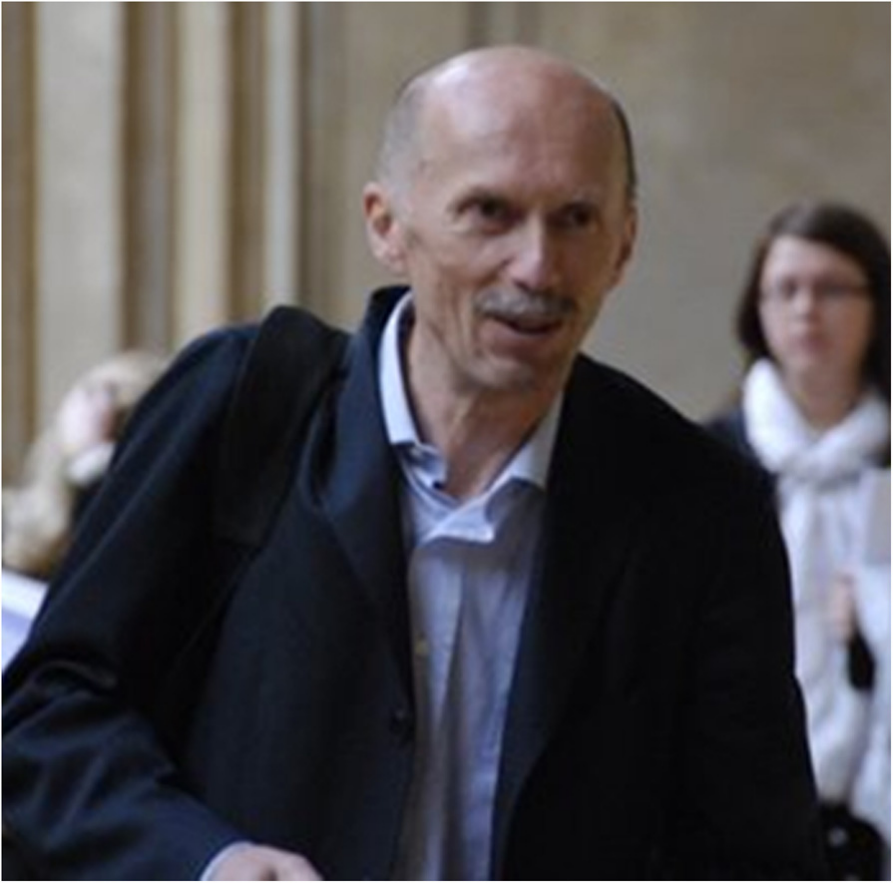
A port trait of Federico Bussolino.

Federico Bussolino (Figure 4), working at the University of Turin, demonstrated for the first time the presence of specific receptors for G-CSF and GM-CSF on the surface of endothelial cells [35-38]. Soldi et al. [39] demonstrated that endothelial cells express the α and ß subunits of GM-CSF-receptor and that GM-CSF is able to activate JAK2 [40]. Moreover, Bussolino showed that GM-CSF induces endothelial cells to migrate, proliferate and release plasminogen activator and is angiogenis in vivo in the rabbit cornea and in the chick CAM assay [35-38,40,41]. Bussolino has also demonstrated for the first time the angiogenic activity of hepatocyte growth factor/scatter factor (HGF/SF), originally identified by Nakamura in 1984 [42].

It is to mention the contribution of another scientist working in Turin, Luca Tamagnone, to the demonstration of the angiogenic activity of semaphorins and plexin [43] and that another Italian scientist, Giovanna Tosato, working in Bethesda, has demonstrated that semaphorin 6A mediated endothelial cell survival by modulating VEGF signaling [44].

Erythropoiesis has been considered the sole physiological action of EPO until EPO and EPO receptor (EPOR) have been found to be expressed in other sites besides kidney, including bone marrow macrophages, neurons, astrocytes, microglia and even oligodendrocytes, cervix, endometrium, ovary, oviduct, and throphoblast cell of the human placenta.

**Figure 5:**
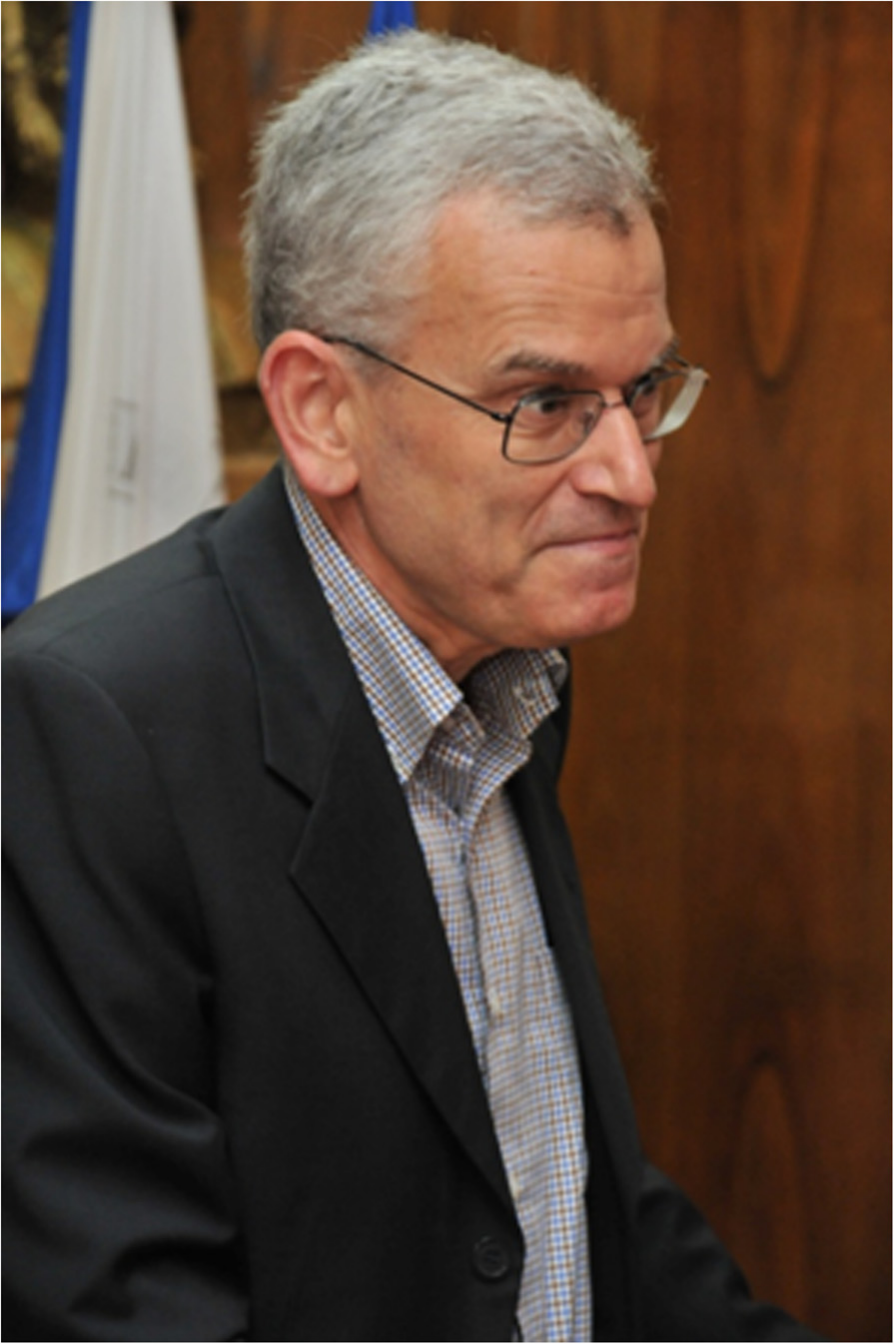
A port trait of Domenico Ribatti.

Moreover, EPO induces endothelial cell proliferation and migration [45,46] and stimulates angiogenesis on rat aortic rings in vitro [47]. In 1999, Domenico Ribatti (Figure 5), working at the University of Bari, demonstrated for the first time that recombinant human EPO (rhEPO) induces a pro-angiogenic phenotype in human endothelial cells [48], including increase in cell proliferation and matrix metalloproteinase-2 production and differentiation into vascular tubes. Accordingly, endothelial cells expressed EPOR that bound to JAK2 and induced its transient activation after rhEPO exposure. In the CAM assay, the angiogenic activity of the rhEPO was similar to that exerted by FGF-2 in the absence of a significant mononuclear cell infiltrate, and endothelial cells of the CAM expressed EPOR. Overall, these data demonstrated that EPO acts as a direct angiogenic factor.

### Concluding remarks

Angiogenesis is controlled by the balance between molecules that have positive and negative regulatory activities and this concept has led to the notion of the angiogenic switch, which depends on an increased production of one or more positive regulators of angiogenesis [49]. Most human tumors arise and remain in situ without angiogenesis for a long time before switching to an angiogenic phenotype, though a pre-neoplastic stage as occurs in breast and cervical carcinomas, which becomes neovascularized before the malignant tumor appears.

Activation of the angiogenic switch has been attributed to the synthesis or release of angiogenic factors, and accordingly to the balance hypothesis, the level of angiogenesis inducers and inhibitors regulates angiogenesis in physiological conditions. This balance is altered in pathological conditions, including chronic inflammations and tumors, as a consequence of an increase bioavailability or activity of the inducer proteins, or reducing the concentrations of endogenous angiogenesis inhibitors.

**Figure 6:**
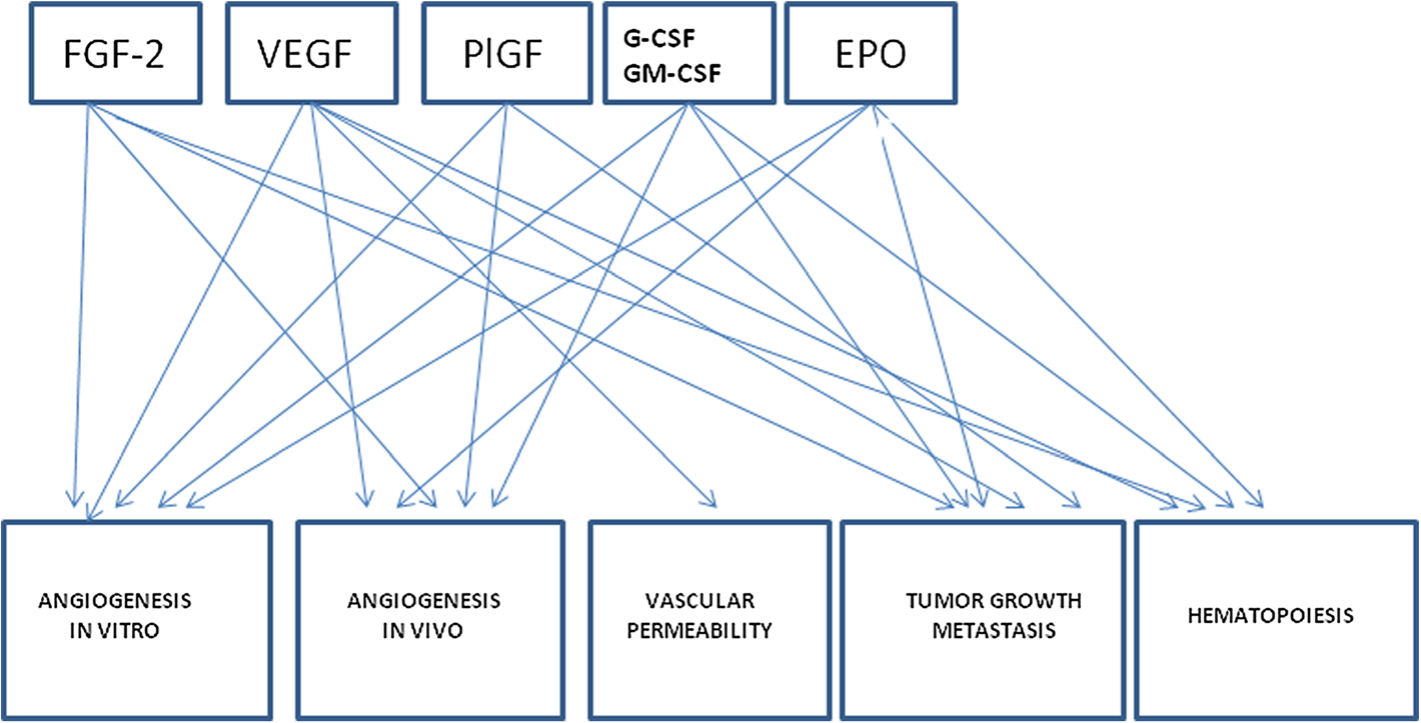
Cross-talk between the biological activities of the principal angiogenic factors.

Angiogenesis is regulated, under both physiological and pathological conditions, by numerous “classic” pro-angiogenic factors, including FGF-2, VEGF, and PlGF (Figure 6). Moreover, evidence has been accumulated that in addition to the “classic” factors, many other “non-classic factors”, including G-CSF, GM-CSF and EPO, play an important role in angiogenesis (Figure 6) [50]. In this article, I have emphasized the important role played by the Italian scientists in the course of the last twenty years to the discovery and characterization of these “classic” and “non-classic” factors.

## Competing interests

The author declares that he has no competing interests.
